# Advances and Applications of Bionic Design and Functional Integration in Underwater Soft Grippers

**DOI:** 10.3390/polym17172408

**Published:** 2025-09-04

**Authors:** Chaoqun Xiang, Hongsen Sun, Teng Wu, Ye Chen, Yanjie Wang, Tao Zou

**Affiliations:** 1 School of Mechanical and Electrical Engineering, Guangzhou University, Guangzhou 510006, China; tao_zhang_tz@163.com; 2 Jiangsu Provincial Key Laboratory of Special Robot Technology, Hohai University, Changzhou 213022, China; 231319010015@hhu.edu.cn (H.S.); 231619010100@hhu.edu.cn (T.W.); yjwang@hhu.edu.cn (Y.W.); 3 College of Mechanical Engineering and Automation, Liaoning University of Technology, Jinzhou 121001, China; jxchenye@lnut.edu.cn

**Keywords:** soft robot, soft underwater gripper, bionic structure design, tactile sensing, grasping-perception integration

## Abstract

This paper systematically reviews the research progress of underwater soft grasping devices in the field of bionic structure, function integration, and tactile sensing technology by drawing on the structural characteristics of marine organisms such as octopuses, jellyfish, and sea anemones (such as suction cups, umbrella-like muscles, and stinging cells). This paper analyzes the inspiration for the design, the application of innovative materials, and the integration of sensing and driving from marine organisms, including a review of soft robotics technologies, such as shape memory alloys (SMA), ionic polymer metal composite materials (IPMCs), magnetic nanocomposite cilia, etc. The research results emphasize that bionic soft robots have the potential for transformation in completely changing underwater operations by providing enhanced flexibility, efficiency, and environmental adaptability. This work provides a bionic design paradigm and perception-driven integration method for underwater soft operation systems, thereby promoting equipment innovation in the fields of deep-sea exploration and ecological protection.

## 1. Introduction

With the ongoing advancements in human science and technology, the continual growth in robotics and automated control theory, and the rising maturity of novel materials and 3D printing technology, a new subset of robots has emerged—soft robots [1]. Soft robots possess a greater number of degrees of freedom, increased flexibility, enhanced safety, and superior adaptation to the environment as compared to conventional rigid robots [2]. Soft robots typically use a single actuator to achieve continuous deformation, and theoretically, the number of degrees of freedom can be infinitely large, enabling the deformation of any curved surface [3]. In contrast, the degrees of freedom of traditional rigid robots are limited by the number of physical joints, and their movement range is constrained by the rigidity of the mechanical structure [4]. The excellent adaptability and safety of soft robots are mainly due to the special nature of their materials, such as graphene-enhanced hydrogel [5], multi-shaped memory hydrogel [6], silicon-based elasticity [7], etc. When in contact with objects, these materials will first undergo a buffer process. In addition, the driving methods of soft robots mostly adopt pneumatic and linear drives [8] and piezoelectric composite materials [9]. In conclusion, soft robots have excellent adaptability, safety, and flexibility. These characteristics enable soft robots to exhibit excellent safety and user-friendliness in interactions between humans and machines, as well as between machines and the environment [10]. Soft grippers have gained significant interest in the area of soft robot technology, particularly in underwater environments. This is due to their remarkable flexibility and adaptation to various materials, allowing them to delicately grasp fragile or irregularly shaped things underwater [11,12,13].

From the beginning of the emergence of soft robots, the design principles of soft robots and bionics have been closely related, principally reflected in the following aspects:

1. Inspired by biological structure: The design inspiration of soft robots generally derives from the structure and behavior of organic species, particularly soft animals; examples include octopuses, jellyfish, and fins [14,15,16,17,18,19]. These animals demonstrate great flexibility, adaptability, and movement, providing a source of inspiration for soft robot design. To concretize their flexibility, adaptability, and movement modes, Table 1 outlines the core biological features of these species and how they inform soft robot design.
2. Application of biomimetic materials: Soft robots commonly employ biomimetic materials, such as silicone [20,21,22], hydrogel [23,24], SMA [25,26,27], etc. These materials have the softness and deformability of biological tissues, so that robots can better adapt to complex situations and execute different jobs.
3. Imitation of motion modes: The motion modes of soft robots are frequently inspired by the motion modes of creatures, such as the soft extension of octopus arms [28] and the crawl of inchworms [29,30]. By replicating the movement of biological creatures, soft robots may attain more flexible and efficient mobility.
4. Biosensing and control: Sensing and control systems of soft robots typically learn from the perception and feedback processes of creatures, such as the lateral line system of fish [31] and human skin [32,33]. These sensory technologies may let the robot detect the surroundings and behave appropriately, boosting its flexibility and intelligence.
5. Integrated perception-execution design: The aim of this design is to allow the soft robot to recognize the target items in the environment and operate these things precisely, with high efficiency, accuracy, and flexibility. This design approach may increase the performance and application range of soft robots.
6. Application of biodynamics: Motion planning and control of soft robots are commonly based on the dynamics concept of animals [34]. For example, motion control algorithms of underwater robots are built by replicating the swimming manner of underwater creatures to provide effective underwater maneuvering and navigation [35].

**Table 1:** The characteristics of marine biomimetic animals.

Characteristic	Sea Anemone	Octopus	Jellyfish
Physical characteristics	The slender tentacles can move with the flow of water, and the body can contract to complete the act of swallowing.	The tentacles can move in a 360-degree circle. The tentacle muscles are well-developed, allowing for switching between rigidity and flexibility.	The tentacles can quickly retract to one-tenth of their original length, and they can also adjust their positions and change the direction of movement.
Grabbing strategy	Using stinging cells as biological toxic injectors, mucus as a physical adhesive, and combining them with the directional contraction of the tentacle muscles to complete the control of the prey.	By wrapping the prey with its tentacles and using suckers to firmly attach and hold prey in place.	Using its tentacles to quickly wrap around an object, and by means of its spicules, hook and contract the tentacles to capture its prey.

**Figure 1:**
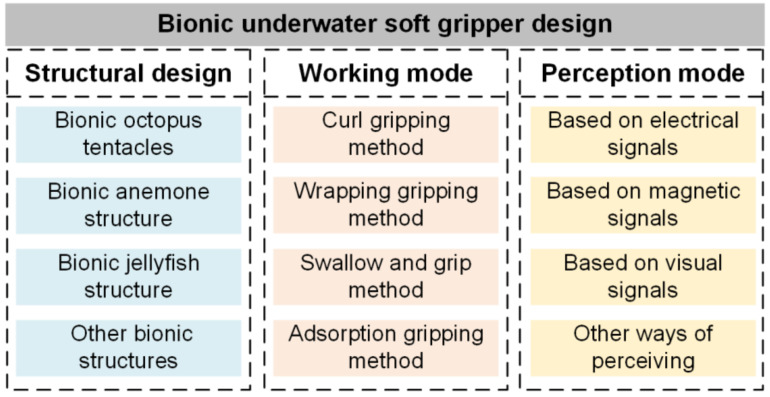
Design analysis of bionic underwater soft grippers.

In recent years, with the continual growth of the ocean, the requirements for underwater grippers are increasing greater and higher. The soft gripper has a distinct advantage underwater; its great adaptability to the environment and the safety of the gripper make it able to fulfill different working conditions and have a broad variety of applications. Researchers have undertaken research on the creation of soft grippers for underwater applications [36]. Soft grippers can better resemble biological structures, because biological structures in nature [36], notably marine biological structures, have been adapted to underwater settings over hundreds of millions of years of development. Therefore, the structural design of the biomimetic underwater soft gripper is inseparable from the study of the structure of marine creatures, and bionics considerably facilitates the advancement of the structure of the underwater soft gripper. As illustrated in Figure 1 below, the fundamental design of the underwater bionic gripper is essentially split into three pieces. First of all, the proper structural design needs to be chosen for underwater work and environmental requirements so that it can be adapted to the underwater environment well and can provide stability throughout the performance of the work. Then, for the item that has to be clamped, a suitable working mode needs to be chosen to guarantee the safety of object clamping and to ensure high work efficiency. At the same time, according to the design requirements of the gripper and the work requirements, the appropriate sensor type needs to be selected for the bionic underwater soft gripper so that it can better perceive the object or the environment and improve the stability of underwater work under the condition of ensuring normal operation.

At present, researchers have analyzed the body structure of octopuses, jellyfish, anemones, and other species that hunt prey by grabbing them and built a series of bionic underwater soft grippers, which have high operating ability and can adapt to most underwater settings. In addition, some of the architecture of researched terrestrial animals is ideal for the creation of underwater soft grippers, including elephant trunks [37], human fingers [38,39], beetles [40], snakes [41,42], and so on. The use of these bionic structures on the gripper may substantially increase job flexibility and safety, and the clamping force in underwater work has been successfully improved.

**Figure 2:**
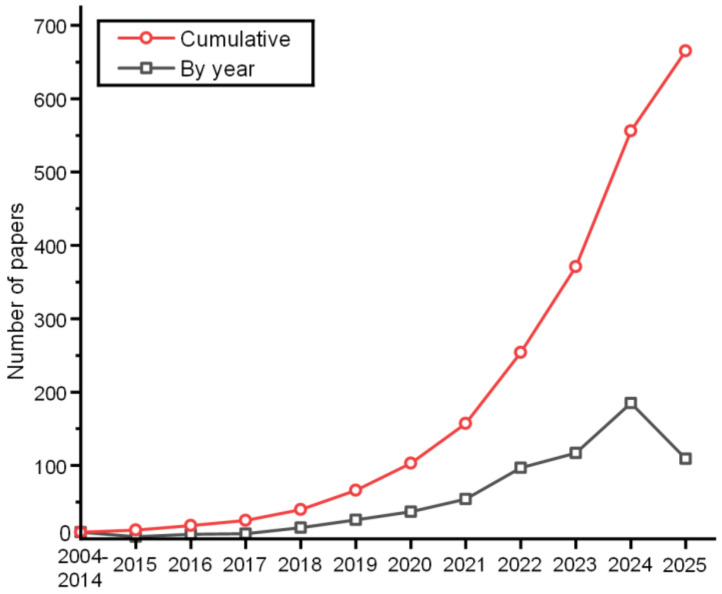
Number of publications per year from Google Scholar in August. The 2025 search terms were <Bionic> AND <soft gripper> AND <underwater>.

To ensure a comprehensive and objective review, we employed a structured approach. Literature retrieval was performed using Google Scholar with strict keyword combinations (e.g., <Bionic> AND <soft gripper> AND <underwater>, <tactile sensing> AND <underwater robotics>) to cover studies on bionic structural design, material applications, tactile sensing technologies, and practical applications. The search was restricted to peer-reviewed articles and conference proceedings published from 2004 to mid-2025. Retrieved studies were screened based on relevance to underwater scenarios, technical novelty, and empirical validation. We then categorized the literature into three thematic clusters: (1) bionic structural design inspired by marine organisms (octopuses, jellyfish, sea anemones); (2) integration of tactile sensing (electrical, magnetic, vision-based) with gripping systems; and (3) application cases in deep-sea exploration, ecological protection, etc. This categorization enabled us to synthesize key advancements, identify research gaps, and propose a ‘Grasping-Perception Integration’ framework. Due to the particular benefits of bionics for the structural design of the gripper, the number of articles connected to the design of the bionic underwater gripper is expanding year by year. Figure 2 displays the number of articles per year from Google Scholar search terms <Bionic> AND <soft gripper> AND <underwater>. It is demonstrated that the bionic structure has a considerable impact on the design of underwater soft grippers.

For the underwater soft gripper, it is not enough to simply have the capacity to grab but also to sense the underwater environment and the geometry of the gripped item in order to adapt its own gripping state and guarantee the stability and safety of the task. At present, common flexible sensors used in soft robotics include resistive, capacitive, electromagnetic, and optical sensors [43,44,45,46,47,48,49,50,51,52], which have certain flexibility and may be incorporated into the soft robot body to achieve the perception of the body condition and gripping stress. In recent years, the cross-modal tactile sensor has had high compatibility with soft robots. It utilizes a soft film to make contact with the item, causes a posture change in cylindrical soil via a form change in the soft film, and then uses a camera to identify the change in the white mark on the convex cylinder to achieve the perception of the object. Tactile sensing can provide soft robots with information regarding surface roughness, object orientation, contact force, and slip measurement [53]. The cross-modal tactile sensor can recognize the depth information and shape information of the item pushed in the contact region and may utilize the camera to record the deformation information created by the object, thereby acquiring the geometric shape of the object [54]. Tactile sensors, as a fusion solution, may play a vital role in locally detecting, controlling, and manipulating things with high sensitivity and spatial resolution, particularly in opaque underwater environments [55,56].

The presence of the cross-modal tactile sensor means the actuator and sensor of the bionic underwater gripper may be fused. However, existing bionic underwater soft grippers still face significant obstacles and restrictions. For example, new intelligent materials and novel bionic architectures have put forth additional criteria for the creation of soft underwater robots. How to construct a gripper that fits the functional criteria is a significant difficulty. Due to the structural properties of soft robots themselves, most sensors used in classic rigid robots are not suited for integration with soft robots. How to create a flexible sensor that fulfills the requirements of soft robots with high precision and excellent compatibility and can be employed underwater is a difficult challenge to be addressed by the designers of bionic underwater soft grippers. Further studies and development are needed to ensure the regulation of underwater gripping power and the adherence of underwater surfaces.

**Figure 3:**
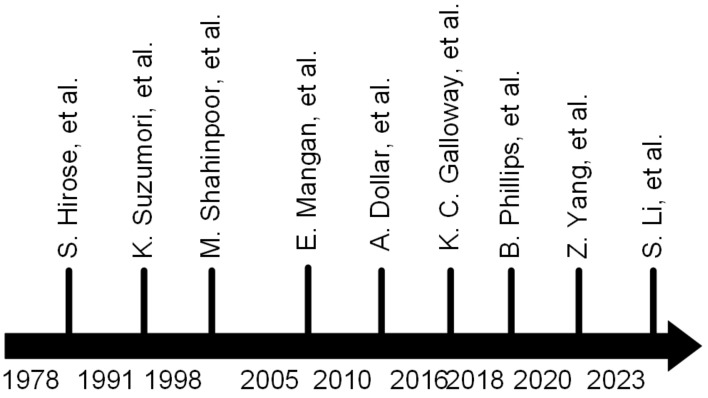
Milestones in the development of soft grippers [13,57,58,59,60,61,62,63,64].

Figure 3 is an abridged timeline of milestones in the development of soft gripper technologies, starting with tendon-driven multilink devices in the 1970s, when Shigeo Hirose and YoJi Umeteni developed a new type of soft gripper that can softly and gently conform to objects of any shape and hold them with uniform pressure. This gripper can be easily actuated by a pair of wires [57]. It can be properly attached to the surface of the item to provide a consistent force to it, strengthening the stability of the holding object. Rapid development of soft grippers took place in the 1990s, with enhanced ideas, materials, and procedures. Soft grippers are no longer powered exclusively by wire; Koichi Suzumori et al. created a flexible electro-pneumatic (or electro-hydraulic) microactuator [58]. M. Shahinpoor, employed Ion-Exchange Polymer-Metal Composites (IPMCs) for actuation [59]. After 2000, in order to increase the working abilities of the gripper, researchers studied the structure of organisms and sought to incorporate it in the structural design of the gripper. Elizabeth V. Mangan et al. designed a gripper according to biological structure, which grips objects via envelopment [60].

The gripper structure created by Aaron M. Dollar can grasp items of diverse forms and has adequate stability [61]. In the 2010s, soft grippers for underwater applications steadily evolved. In order to overcome the issue of the delicate manipulation and sampling of sensitive species on seafloor reefs, soft grippers designed by Kevin C. Galloway and others can have various grasping modes to ensure the safety of the gripped objects [13]. Brennan T. Phillips and colleagues developed a synchronized gripper that utilizes a remote-controlled glove to operate an underwater soft gripper, ensuring operational precision [62]. The above examples prove that soft grippers have obvious advantages in underwater work. In order to ensure the precision of the gripper, researchers have examined the integrated perception-gripper design to ensure the safety and stability of the task. Zeyi Yang et al. developed a soft gripper that could sense its own condition in real time using an embedded sensing mode employing optical fibers [63]. A soft gripper termed JamTac was developed, which offers high-resolution tactile perception, a broad detecting surface, and integrated sensing grabbing capacity that can ask for and grasp in low-visibility environments [64].

In order to make better use of bionic soft grippers in underwater work applications, an in-depth knowledge of the grippers’ concept, design, manufacturing, and testing is necessary. This study systematically reviewed the structural design of underwater biomimetic grippers, analyzed the structural characteristics and functional mechanisms of different biological prototypes (such as octopuses, jellyfish, sea anemones, etc.), innovatively proposed an integrated design framework of perception and grasping, and through cross-modal analysis based on the principle of tactile sensing, integrated the adaptive characteristics of resistive, capacitive, magnetic field, and visual sensors to solve the tactile perception bottleneck of underwater soft grippers in complex fluid environments. The use of underwater tactile perception in bionic underwater grippers is examined in order to present a new concept for the integrated design of bionic underwater grippers. This survey tries to bridge the gap and fulfil this critical requirement. The rest of this article is arranged as follows: Section 2 presents the biological structures usually utilized for reference in the creation of biomimetic underwater soft grippers and the grippers created according to these structures. Section 3 presents many types of tactile sensing and their operating principles. Section 4 presents the primary application sectors of underwater soft clamping. Section 5 examines the design, sensing, and use of the biomimetic underwater soft grippers and analyzes existing drawbacks. Section 6 summarizes this article.

## 2. Structural Design of Bionic Underwater Soft Grippers

The organic structure of nature offers an excellent reference for the design of soft robots [2], which allows them to better adapt to the underwater environment and accomplish more complex underwater activities. The flexibility of organisms living in the ocean has influenced the construction of underwater robots. Marine creatures constitute an excellent aid and inspiration for the structural design of underwater soft grippers. Marine animals such as octopuses, jellyfish, and sea anemones are key reference items for the design of bionic soft grippers. Based on the biomimetic analysis of the grasping mechanisms of these marine creatures, their unique structures provide crucial inspiration for the design of soft grippers, as they possess unique characteristics and advantages in underwater environments. For example, octopuses achieve efficient search, stable grasping, flexible manipulation, and precise transportation through their stretchable muscular tentacles and highly complex hierarchical suction cup systems. Jellyfish and sea anemones mainly use their arrays of tentacles covered with fine spines or adhesive secretions to passively capture prey. By deeply mimicking the morphological structures and functional principles of these organisms—particularly the active extension and adhesion capabilities of octopus tentacles and the passive winding and adhesion characteristics of jellyfish/sea anemone tentacles—the developed biomimetic soft grippers can integrate and expand their functions. This design gives the grippers the ability to operate in diverse modes, including adaptive grasping, non-destructive transportation, fine touching, and passive capture. Ultimately, this biomimetic strategy inspired by multiple species significantly enhances the flexibility, environmental adaptability, and task universality of soft grippers. Therefore, the core content of this section focuses on the design principles of soft gripper structures that integrate the biomechanical characteristics of octopuses, jellyfish, and sea anemones.

### 2.1. Octopus-Inspired Underwater Soft Gripper

As a very magical and fascinating marine creature, the octopus has a soft and elastic body, and its tentacles are composed of many flexible segments, each of which is equipped with suction cups, so that it can flexibly adapt to objects of different shapes and surfaces and achieve accurate grasping and manipulation [65]. Thus, among many species, octopuses provide fresh concepts for developing robots capable of examining and grasping in confined and disorganized environments [66].

**Figure 4:**
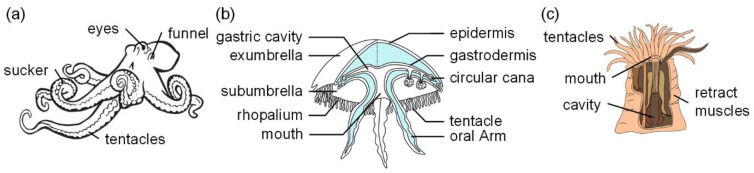
Biological structures. (**a**) Octopus structure. (**b**) Jellyfish structure. (**c**) Sea anemone structure.

Inspired by the biological structural characteristics of an octopus (shown in Figure 4a), a soft robotic arm was designed with suction cups to increase grasping tasks in limited areas [66]. The robotic arm contains soft and firm silicone rubber materials and driving tendons, allowing it to travel through air, water, and oil, and grab things of diverse forms, enabling efficient twisting around objects with diameters up to 30 mm. The suction cup is propelled via a fluid channel to adapt it to a limited region of unknown form. The design of this soft robot arm relies on the form and function of the octopus arm and leverages soft robot technology to achieve great flexibility and operating capabilities. The findings from testing illustrate the efficiency of the manipulator in grabbing complex items and operating in varied contexts.

However, suckers for soft robotic arms are generally intended to adsorb specific forms of surfaces, such as smooth metal or non-porous materials. As a consequence, its adsorption ability may be restricted when presented with items of various materials and textures. Especially for rough, porous, or damp surfaces, the adsorption action of the sucker may be considerably diminished. In addition, while working in a restricted location, the robotic arm may be vulnerable to environmental interference, such as vibration, temperature changes, or variations in air pressure. These variables may contribute to the instability of the adsorption force of the suction cup, therefore reducing the precision and dependability of the gripping work.

Therefore, researchers conducted studies to address the aforementioned issues. Kansai University in Japan invented a vacuum sucker soft gripper that imitates octopus suckers. Elastomers are employed as the major functioning portion of the gripper. Multiple tiny suckers are positioned on the gripper surface, and each sucker may act independently to adsorb items by creating negative pressure, which can grab a cylinder with a diameter of 20 mm [67]. Hosei University in Japan created a flexible continuous arm manipulator inspired by octopus morphology and intelligent behavior. This manipulator is mostly constructed of soft silicone rubber material, which has the characteristics of high deformation and flexible movement. Various motions may be created by inserting a sinew-like driving string in the arm, which can grip a 40 × 40 × 190 mm banana [68]. A bionic flexible gripper operated by shape memory alloy (SMA) wire was proposed by Northeast Forestry University. Its arm used SMA wire to imitate the fiber of an octopus arm, which was placed it at the base of the robot arm, parallel to its central axis [69]. The Institute of Agricultural Facilities and Equipment of Jiangsu Academy of Agricultural Sciences built a three-finger flexible bionic robot gripper inspired by octopus tentacles. Its hand components are formed of finger claws and finger teeth. Using a flexible pneumatic drive, it accomplishes two motion modes comparable to octopus tentacles, gripping and relaxing. The experimental results show that the 100.0 g bionic gripper can load an apple with a weight of 246.5 ~ 350.0 g and a diameter of 69.0 ~ 99.0 mm, and the success rate was 100% in 90 grabbing trials [70]. Zhejiang University examined the design of an octopus heuristic adaptive soft gripper based on 4D printing technology [71], which used a double-layer structure made of a semicircle and three rectangles to simulate the network and tentacle-like behavior of an octopus, respectively. This design makes the gripper have low stiffness at high temperature and can accomplish the adaptive gripping of items. After cooling, it has high rigidity to ensure the stability of grabbing. The driving procedure is as follows: When the soft gripper is heated, the difference in CTE between the several layers within it will cause the structure to bend or twist. This deformation process resembles the movement mechanism of octopus tentacles, enabling the soft gripper to adaptively modify its shape to adapt to the grabbing demands of diverse items, and is capable of gripping eggs with a maximum radius of 30 mm. Although the effectiveness and reliability of this soft gripper in grasping objects of different shapes and sizes are high, the design and manufacturing process is extremely complex due to its advanced 4D printing technology. The Intelligent Bionic Design Laboratory of Peking University designed a soft robot gripper with adaptive underwater grasping and sensing [72]. The gripper core is a 3D-printed linkage mechanism with tubular bellows inside. The bellows can deform when pushed, which forces the silica-cast soft suction cups to expand and compress. At the same time, a number of hollow nozzle arrays are equally dispersed on the suction cup of the gripper, which can increase the adsorption force of the suction cup and adapt to the gripping demands of diverse surfaces. In addition, the driving mode of the gripper mostly relies on the pneumatic drive. Specifically, it employs a number of independent pneumatic chambers, and by infusing air into these chambers, the drive of the soft finger is produced. This driving mechanism allows the soft fingers to create an array, which enhances the flexibility and mobility of the gripper while grabbing items.

As can be seen from the previous studies, soft grippers that mimick an octopus mainly imitate the tentacle or the suction cup structure on the tentacle, the biological structure characteristics of which are shown in Table 2. By imitating these structures, the soft gripper has high adaptability and flexibility, but the control complexity is also high, as is the sealing performance of the device. The advantages and disadvantages of octopus-inspired soft grippers are presented in detail in Table 2. All in all, these studies provide a new idea and method for the bionic design of underwater soft grippers, which are expected to play an important role in underwater operations, ocean exploration, and other fields.

### 2.2. Jellyfish-Inspired Underwater Soft Gripper

Jellyfish are predatory animals of the sea. They consist of a transparent, gelatinous material. Jellyfish have tentacles with pallets and cells that are utilized for hunting, defense, and detecting the surroundings. Jellyfish normally have a spherical or umbrella-like body, which comprises circular muscles and radial muscles, which are found in the center layer of the umbrella (as shown in Figure 4b). When the jellyfish desires to move, both the annular and longitudinal muscles apply effort at the same time. The contraction of the annular muscle causes the circumference of the umbrella to tighten, while the contraction of the longitudinal muscle causes the height or radius of the umbrella to decrease [83,84]. Jellyfish utilize stinging sacs on their tentacles to stab or kill animals and then bring food to their mouths and digesting sacs [85].

According to the biological properties of jellyfish (as indicated in Table 2), their dome structure is a bionic orientation of the soft gripper. Temple University examined the design of a multipurpose soft dome drive, which consists of a circular double-layer structure. When air is inflated into the spiral air channel, the radial expansion of the top layer generates a mismatched deformation between the top and bottom layers, resulting in a three-dimensional dome-like appearance. After decompression, the dome form reverts to its planar double-layer structure [73]. A research team from Dalian University of Technology has come up with a wrapped soft gripper inspired by the idea of the jellyfish drive. The gripper comprises four precisely built bionic soft actuators, which can imitate the bending motion of the jellyfish’s central and longitudinal muscles [15]. These soft actuators resemble the musculature of jellyfish in structure, and by manipulating the pressure fluctuations inside them, they can bend and stretch. When the pressure within the actuator rises, the actuator will bend, similar to the contraction of the jellyfish ring muscle. When the pressure is dropped, the actuators lengthen, similar to the relaxation of the longitudinal muscle of a jellyfish. The findings of finite element analysis reveal that the bionic soft actuators can accomplish bending akin to jellyfish muscles. Finally, the bionic soft gripper is connected to the KUKA robot to capture the target item. The experimental findings reveal that the suggested bionic soft gripper can reliably grasp things such as seafood and eggs with smooth surfaces. The soft doming actuator represents a significant advancement in multifunctional design for underwater applications. It stands out for its versatility, adaptability, and simplicity compared to traditional underwater grippers. However, it may not match the mechanical strength of more specialized, rigid grippers, particularly in tasks requiring high force or precision. Therefore, its use is most advantageous in scenarios where flexibility, energy efficiency, and multifunctionality are prioritized over sheer mechanical power.

Nevertheless, the elastic materials used in the wrap soft gripper are not capable of enduring challenging conditions or frequent high-intensity gripping actions over an extended period. Consequently, this leads to a decline in performance or potential harm. Table 2 displays the grabbing capabilities, as well as the advantages and drawbacks of the soft gripper that is inspired by jellyfish. Hence, the advancement of novel materials with enhanced durability and stability is crucial for enhancing the performance of these robots. Simultaneously, the soft grabber’s flexibility must be enhanced to accommodate items with varying forms, sizes, and weights. This task may require the use of more sophisticated control systems and superior sensor technologies.

### 2.3. Sea Anemone-Inspired Underwater Soft Gripper

As a marine creature with effective catching abilities, the column structure of the anemone is formed of a foot disk, a mouth disk, and a body wall. Numerous tentacles of the anemone are spread on the oral disk, and the mouth is positioned in the middle of the oral disk and linked with the throat. This body form and its effective predation mechanism are crucial determinants for the survival of anemones in the lengthy evolutionary process [86]. Anemones are carnivorous organisms that utilize their tentacles to catch prey. Once the prey is hurt by the stinging cells on its tentacles, the anemone gently draws it into its mouth and consumes it with its mouthparts. By emulating this predatory behavior, anemone imitator soft grippers [74] integrate sensory and multimodal gripping capabilities.

As illustrated in Figure 4c, the biological structural properties of anemones offer great research significance in the design area of underwater grippers. For example, Southwest University of Science and Technology produced a gripper prototype based on the predation behavior of sea anemones [74], which can grasp objects of different geometric shapes, weights, and materials, proving the adaptability of its structure in various environmental applications and providing an alternative way to design adaptive grippers. Based on the features of anemone tentacles shrinking in the face of danger or food capture, Yantai University successfully built a bionic anemone gripper, which employs a light-driven grasp contraction mechanism [75]. The gripper device successfully integrates optical drive technology with magnetic drive technology, which gives a novel concept for a new driving mode for underwater grippers. The University of Bristol developed a bistable soft gripper based on the structure of anemone [16], which can effectively grip objects of various shapes, and the grasping success rate of objects of 4 mm to 10 mm size is more than 90%, which highlights the potential application value of using anemone structure in the design of underwater grippers.

The anemone-inspired soft robot designed according to the capture characteristics, motion characteristics, and structure of the anemone has good environmental adaptability and capture success rate. However, the capture characteristics, motion characteristics, and structural structure of the anemone have not been integrated with an underwater gripper. Therefore, the development of a gripper solution that is suitable for underwater environments can promote the development of underwater robot technology and can also bring creative solutions for future sectors such as ocean engineering.

### 2.4. Other Bionic Soft Grippers

Besides the soft grippers based on octopuses, jellyfish, and anemones, there are also soft gripper designs based on elephant trunks [77], chameleons [87], and geckos [78]. For example, a research team from Harbin Institute of Technology created the shape of novel PAMs based on the distribution and mobility of the muscle fibers in elephant trunks [77]. Different materials and manufacturing procedures were employed to approximate the flexibility and bending capabilities of the elephant trunk. PAMs utilize constructions such as braided tubes or reinforced flexible frames to enable multi-directional bending and twisting. In gripping experiments, it was able to grip a full bottle of water with a diameter of 64 mm and a hot melt glue gun (248 g, 200 mm long) by holding the head.

Furthermore, there are many such underwater soft grippers, but they still need to be improved, which may become a future research path for underwater soft grippers. For example, Harvard University developed an underwater gripper, which employs soft robotic technology to delicately handle and sample endangered species on deep reefs. During the test, the gripper was able to grip a 50.8 mm diameter tube. However, it was unable to grab underwater detritus [13]. A prototype of a four-finger gripper based on a soft actuator was developed at Beihang University and demonstrated excellent grasping capability in amphibious environments, especially for soft and fragile objects. In the grabbing experiment, the gripper could successfully grasp a sphere with a diameter of 170 mm, a cup with a weight about 250 g, and a screw with a diameter of 3 mm. However, the gripper could be subject to large eddy currents and flow resistance when operating in running water [76]. The Technical University of Cartagena has designed and manufactured a simple, low-cost, and easy-to-deploy soft gripper for underwater operation, which uses silicone rubber to cover the soft actuator, is additively manufactured in flexible thermoplastic, and increases underactuations in the contacts by distributing the grasping force to suit the geometry when maneuvering. It provides high sensitivity and mechanical dependability. The experimental results show the results of the testing of the soft gripper at different internal pressures (120 kPa to 500 kPa). Under these pressure conditions, the performance of the manipulator was evaluated by observing the displacement of the non-end-effector on the *X*- and *Y*-axes. At each pressure level, the experimental trajectory of the end-effector over time was measured. The data showed that the higher the pressure, the greater the displacement, suggesting that the manipulator was able to grasp objects more efficiently with increased internal pressure. The soft gripper gripped a plastic lobster attached with a rope to a 250 g weight., However, flexible thermoplastics may be affected by durability issues [79]. Similarly, Northeastern University designed a bionic soft gripper for underwater operations whose clamping action is realized by driving water or air, and by adjusting the fixed position of the claw on the flange, it can handle target objects of different sizes, the size of 175 × 130 × 38 mm in the grasping experiment.

A plastic lobster with a weight of 114.31 g can be grabbed efficiently; however, there are still limits in the grip of fine things [38]. Therefore, the University of Calabria, Italy, has built a novel soft-controlled robotic underwater gripper for the delicate hold of underwater items. Its gripper employs two neutral buoyant particle jamming pads to securely and reliably grab items of any form, including delicate and/or large targets; for example, in the grasping experiment, the gripper pulled a 4.5 kg solid rock (with a diameter of 20 cm) out of the water and held it in the air [80]; however, it was difficult to hold flat items anchored to the bottom of the water. Beijing University of Technology has developed a hydraulic flexible gripper with a three-finger configuration to overcome the issue of the low adaptation of current underwater grippers. Driven by water hydraulic pressure, the gripper can achieve flexible gripping, has simple construction, high pressure, excellent adaptation to water environments, powerful anti-interference ability, and easy control. However, it largely decreases the effect of aquatic environment elements via structural design, which limits the structural design of underwater soft grippers [81].

It can be seen from previous research that the current underwater soft gripper has certain limitations in the gripping of underwater objects (as shown in Table 2). In regard to the influence of underwater factors (water flow, water pressure and) on grasping performance, the water dynamic characteristics are mainly solved through structural design and materials. In addition, the previously described underwater soft gripper does not evaluate the impact of water film on the grabbing performance. The gripper accomplishes the gripping of an item by the action of friction; however, in the underwater environment, when the gripper touches the object, there is a certain pressure. This pressure forces the surrounding water into the small space between the tentacle and the item, generating a thin layer of water. This layer of water film has particular lubricating features, which minimizes the friction between the tentacle and the gripped item and impacts the gripping performance of the bionic underwater soft gripper. Therefore, the absence of a method to construct the grasping force compensation mechanism under the effect of underwater elements leads to the instability of the gripping force of the soft gripper, which renders the soft gripper unable to grab items securely in the underwater environment.

## 3. Underwater Tactile Sensing System

### 3.1. Integrated Design for Underwater Sensing and Grasping

For the underwater soft robot, it is not enough to merely have the capacity to grasp, but it also needs to detect the underwater environment and the geometry of the gripped item in order to change its own grasping state and guarantee the stability and safety of the task. How to improve the safety and stability of interaction between underwater robots and objects under the influence of complex underwater environments has high academic and application value, and improving the interaction performance of the end effector of underwater robots in complex water environments is a key factor in solving this problem, especially in the case of interaction with soft biological tissues under extreme water flow, water pressure, and low visibility [80]. In order to increase the interaction performance of the end effector in a complex aquatic environment, an innovative solution is to merge the sensing and clamping system to produce an integrated sensing and clamping design. This design not only allows the underwater soft robot to detect the underwater environment and the features of the gripped item in real time but also automatically changes the grasping state according to the perceptual findings to guarantee the stability and safety of the task. By merging sensor technology and clamping systems, soft underwater robots can cope with diverse difficult circumstances more intelligently, eliminate manual intervention, and enhance job productivity. Therefore, the combined sensing and clamping design will make substantial advances in the development of underwater soft robots, making them play a more crucial role in underwater operations. However, in order to implement the integrated sensing and clamping structural design of the underwater soft gripper, it is necessary to evaluate whether the sensor is appropriate for use in water and if the sensor can be integrated on the soft gripper. Since the soft gripper is formed of soft materials and prone to substantial deformation, the integrated sensor cannot influence the deformation of the soft gripper.

In addition, due to the complex underwater environment, underwater sensors can sense the external environment and its own state as feedback signals for interaction between the end effector of underwater robots and objects, such as underwater acoustic sensors [88], underwater optical sensors [89], underwater electromagnetic sensors [90], etc., but underwater acoustic sensors are susceptible to acoustic multipath effects and acoustic reverberation [11,91]. Underwater optical sensors will impair visibility and contrast owing to underwater light absorption and scattering [92]. Changes in the quantity of electrolytes in water impact the strength of electromagnetic fields, which dramatically lowers the perception accuracy of underwater sensors. Moreover, the effective distance is low, and interference and overlap can easily occur when numerous sensors are operating [93]. These underwater elements have a detrimental effect on sensor performance and accuracy, resulting in their adaptation to the underwater environment and the criteria of underwater soft grippers not being fulfilled, and these sensors are not easily combined with underwater soft grippers. In recent years, cross-modal tactile sensors have had strong compatibility with soft robotics. Tactile sensors can offer information about surface roughness, object orientation, contact force, and slip measurement for soft robots [53]. Therefore, haptic sensors as a fusion solution can surely play a vital role in locally detecting, controlling, and manipulating objects with high sensitivity and spatial resolution, particularly in opaque underwater settings [55,56]. In addition, tactile sensor materials are mostly flexible and can be highly compatible with soft grippers and realize the perception of objects without affecting performance. The following is an overview of tactile sensors based on electrical signals, magnetic fields, vision, and other tactile sensors, and an analysis of their principles, performance, advantages and disadvantages, and the problems faced by such tactile sensors in water, so as to provide certain suggestions for future research on underwater tactile sensing technology and the integrated design of sensing and grasping.

### 3.2. Tactile Sensors Based on Electrical Signals

**Figure 5:**
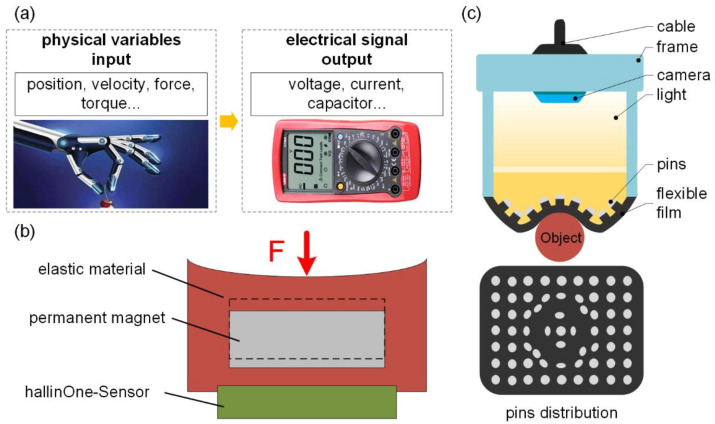
Tactile sensor principle. (**a**) Tactile sensor based on electrical signals. (**b**) Tactile sensors based on magnetic fields. (**c**) Tactile sensors based on vision [130].

Tactile sensors based on electrical signals are a type of sensor that can detect and measure information such as surface features, forces, or object deformation and convert it into an electrical signal output (its sensing principle is depicted in Figure 5a). These sensors are typically employed to imitate the human sense of touch to provide tactile perception and control in sectors such as robotics, automation systems, or medical equipment. At present, typical tactile sensors based on electrical impulses are based on resistance, capacitance and voltage. This sort of tactile sensor has the benefits of high sensitivity, significant dynamic range, and high frequency response, as illustrated in Table 3.

**Table 3:** The benefits and drawbacks of tactile sensors and underwater working conditions.

Sensor Technology	Signal	Advantage	Disadvantage	Underwater Working Condition	Reference
Tactile sensors based on electrical signals	Capacitance value, resistance value, charge value	High sensitivity, large dynamic range, high frequency response	Sensitive to noise, poor repeatability, hysteresis, high power consumption, complex measurement circuit	Easily influenced by the dielectric constant and conductivity of water	[94,95,96,97,98,99,100,101,102,103]
Tactile sensors based on magnetic fields	Magnetic field intensity	High sensitivity, strong adaptability, multi-touch	High complexity and easily interfered with by external magnetic fields	Susceptible to water conductivity	[53,104,105,106,107]
Tactile sensors based on vision	—	High resolution, versatility, flexibility and fast response	High computational complexity and easily affected by the environment	Easily affected by water flow and pressure	[32,108,109,110,111,112,113,114,115,116,117,118,119,120]
Other tactile sensors	—	Fast response speed, high resolution, and high sensitivity	Vulnerable to environmental impacts, complex manufacturing, high cost	—	[121,122,123,124,125,126,127,128,129]

The University of New South Wales has created a novel shear force sensor design. The sensor is designed for usage in robotic hands for the measurement of shear stress at the tactile interface [94]. The experimental findings suggest that the standard deviation for complete sensor range is 1.35 × 10^−15^, which is considerably greater than the standard deviation at other load positions. The largest standard deviation, for each force value, within a range of ±2 N, is 4.28 × 10^−16^ F. It can be concluded from the experimental findings that the sensor is able to transduce changes in displacement and shear force with rather good repeatability. Therefore, the sensor has great sensitivity and accuracy. In addition, the sensor is built utilizing a printed circuit board, which has the benefit of cheap cost and simple mass manufacturing. Through its characteristics, we believe that the shear force sensor developed at the University of New South Wales can be integrated into the haptic interface of a soft gripper to detect shear stress on objects in an underwater environment. The sensor is able to provide accurate shear force data during relative motion between the gripper and the object, which is particularly important for gripping soft and fragile objects. By accurately measuring the shear force, the gripper is better able to adjust its gripping force and angle to avoid damage to the object. National Tsing Hua University has successfully shown a revolutionary three axis polymer tactile sensor with built-in piezoresistive sensors. The sensor comprises a polymer membrane and four sensing cantilevers with piezoresistors on both the top and side walls to measure the in-plane and out-of-plane loads. By inserting the nanoparticles into the polymer, the stiffness of the membrane can be readily modified. The results reveal that the rigidity of the PDMS membrane rises from 1.32 to 479.25 MPa after introducing Co particles of PDMS/Co = 10/1. Meanwhile, it rises from 1.32 to 479.25 MPa after adding co-particles of PDMS/co = 10/1. The sensitivity of the tactile sensor is greatly lowered. However, the maximum acceptable load of the tactile sensor is increased [95]. The haptic sensor’s ability to measure in-plane and out-of-plane loads makes it ideally suited for use as an end-effector in soft-bodied grippers. By measuring forces in different directions, the sensor can help the gripper to be more flexible in manipulating objects in complex underwater environments. The stiffness of the polymer membrane can be optimized by the adjustment of the nanoparticles, which not only improves the mechanical durability of the sensor but also adapts it to the needs of different gripping tasks. The Feinstein Institute for Medical Research created flexible dome- and bump-shaped piezoelectric tactile sensors utilizing the PVDF-TrFE copolymer. The tactile sensors constructed employing these polymer microstructures demonstrate high sensitivity, which can sense forces as small as 40 mN for bump-shaped sensors and 25 mN for dome-shaped sensors [96]. Because the sensor material is the PVDF-TrFE copolymer, the piezoelectric characteristics of the sensor may be severely altered when it operates in a high-temperature environment, resulting in the instability or distortion of the sensor output signal. At the same time, the material may become brittle and rigid at low temperatures, reducing the flexibility and endurance of the sensor. The flexible piezoelectric tactile sensor is capable of detecting very small force changes due to its high sensitivity, allowing the gripper to excel when handling small or sensitive objects. They also provide fast force feedback in underwater environments, which is particularly important for tasks that require precise maneuvering, such as biological sample collection or tiny object grasping. Sarkar, D., et al. developed a lightweight, flexible, and integrated multimodal tactile sensor, which was integrated on VR gloves to enhance users’ interactive experience in Virtual Reality (VR) and remote robot operation. It can sense the user’s pressing force when using VR gloves and control the virtual manipulator for operation. Users had an average success rate of 91.67% for grasping virtual objects (cubes, spheres, cylinders), addressing limitations in haptic feedback and motion tracking [131].

It can be seen from the previous studies on tactile sensors based on electrical signals, as shown in Table 3, that such sensors have the advantages of high sensitivity, large dynamic range, and high frequency response, but they have disadvantages such as sensitivity to noise and the environment, poor repeatability, high hysteresis, high power consumption, and complex measurement circuits. In addition, while operating in a water environment, owing to the high dielectric constant and conductivity of water, it is possible to interfere with the signal of capacitive and voltage tactile sensors. For resistive sensors, the presence of water could affect the resistance value of the resistive material, therefore reducing the accuracy and stability of the sensor. At the same time, water molecules may build a liquid film on the surface of the sensor, which can also cause the sensor’s measurement signal to be blocked or distorted.

### 3.3. Tactile Sensors Based on Magnetic Fields

Tactile sensors based on magnetism are another sort of tactile sensor that could replicate the mechanosensorial receptors in human fingertips. Robustness and a lack of mechanical hysteresis are benefits of such tactile sensors (its perception theory is depicted in Figure 5b). Because of these benefits, magnetic field-based tactile sensors have gained a lot of interest in the research sector.

Ledermann et al. presented a magnetic tactile sensor employing a commercial 3D Hall sensor, AS54xx of the Fraunhofer Institute for Integrated Circuits (IIS) in Erlangen, Germany, and incorporating a permanent magnet in the elastic material that covers that AS54xx [104]. The functional structure of the detecting section that perceives the applied forces of their tactile sensors is comparable to the construction of conventional capacitive tactile sensors. The elastic material will deform when external forces are applied, which can affect the location of the permanent magnet. The AS54xx can measure the change in the magnetic field vector so that the magnitude and direction of forces can be acquired. However, the diameter of the circular PCB in which the 3D Hall sensor AS54xx is created is 9 mm, and the complete sensor prototype is built in a silicon pad with a diameter of 16 mm, which restricts the actualization of a high spatial resolution [53]. Alfadhel et al. have presented a form of tactile sensor employing magnetic nanocomposite hair-like cilia, which can sense minute surface texture changes [105,106]. However, because the tactile sensor employs a nanocomposite material composed of very elastic polydimethylsiloxane and iron nanowires, the production method of this material requires careful control of the composition ratio of the material, the size and distribution of the nanowires, as well as features such as the elasticity, corrosion resistance, and thermal stability of the material. These criteria make the material preparation process complex and difficult to regulate. In addition, Wattanasarn et al. presented a three-dimensional magnetic tactile sensor employing flexible induction coils embedded in elastomeric substrates [107]. The experimental findings revealed stronger stabilities in the readout signals, with an average SD of 0.109 mV, than a capacitive measurement-based approach. In addition, the flexible tactile sensor is resilient to parasitic interference, sensitive, and easy to produce for diverse domains of applications. However, the structural design and signal processing mechanism of the tactile sensor are exceedingly complex. In actual applications, the presence of numerous interference elements, such as electromagnetic interference and mechanical vibration, may lead to signal distortion or confusion, therefore decreasing the measurement accuracy of the sensor.

As can be observed from the previous study, the magnetic field-based tactile sensor does not need to physically contact the target item and can carry out touch detection without hurting or disturbing the object, its detection sensitivity is high, and its adaptability is excellent. Moreover, these magnetic haptic sensors can enhance the soft gripper’s force sensing and localization capabilities in underwater environments as well as enhance surface texture detection capabilities. In terms of force sensing and positioning capabilities in underwater environments, this is important for soft gripper tasks that require precise control of gripping force and direction during underwater operations. By integrating such sensors, soft-bodied grippers can more accurately adapt their operation to the complexities of the underwater environment. In terms of enhanced surface texture detection, the gripper can sense small surface texture variations, which is useful when identifying and manipulating fine-structured objects in underwater environments. With this sensor, the soft gripper can thus better detect and manipulate objects with different surface characteristics, such as rocks, marine life, or shipwrecks, in the underwater environment. However, the design and construction of magnetic field-based tactile sensors is difficult, and they are susceptible to being disturbed by external magnetic fields, especially while operating in a water environment. Since water is a material with high electrical conductivity, it may bring extra magnetic field interference, which may compromise the accuracy and stability of the magnetic field-based tactile sensor. The individual benefits and drawbacks are listed in Table 3.

### 3.4. Tactile Sensors Based on Vision

The above sensors are often affected by a variety of factors during underwater operation, including but not limited to temperature fluctuations, variations in environmental conditions, and the presence of magnetic fields [108,109,110], which may negatively affect the performance and accuracy of the sensors, resulting in their adaptability in underwater environments that are inconsistent with the requirements of underwater soft grippers. Tactile sensors based on vision demonstrate high adaptation to aquatic ambient conditions.

A vision-based tactile sensor, also known as an optical tactile sensor, generally consists of three basic components: a camera imaging system, a light source structure, and a flexible contact layer with markings [111]. Its perceiving concept (as illustrated in Figure 5c) is to evaluate visual data by employing computer vision techniques, such as surface texture analysis, geometric form reconstruction, and depth perception. By evaluating the visual aspects of an item, such as shadows, high brightness changes, and texture details, it is feasible to deduce the tactile attributes of the object, such as hardness, surface roughness, and form.

Early vision-based tactile sensors were almost all designed using the principle of the total internal reflection of light, such as the optical waveguide tactile sensor proposed by the National Institute of Industrial Science and Technology of Japan in the 1990s, which uses optical fibers to introduce lighting into the waveguide. When an item touches the elastic shell, the elastic shell contacts the optical waveguide in such a way that the light is dispersed and then caught by the camera. For a contact item with a given form, its normal vector can be computed; thus, the sensor can feel both the contact location and the normal force during the contact event [112]. At the same time, there are other optical and tactile sensors built based on the idea of marker tracking, such as Kamiyama et al., which are used to detect the distribution of three-dimensional force vectors [113] and dubbed Gelforce sensors [114,115]. Such sensors are constructed of a transparent elastomer coated with black paint, a clear acrylic plate, a blue and red marker ball placed within the elastomer, and a color CCD camera. The CCD camera catches the movement of the marker produced by the skin pressure of the item contacting the elastomer and calculates the displacement of the red and blue markers based on the elastic theory to obtain the amount and direction of the contact force.

In addition, MIT employed structured light and photometric stereoscopic vision algorithms to identify tiny textures of touch surfaces using GelSight tactile sensors [116]. Mike Lambeta et al. built a deep reinforcement learning algorithm on the DIGIT tactile sensor to combine high-dimensional information provided by contact and obtain useful touch feedback [117]. The University of Bristol has created the TacTip cross-modal haptic sensor, which uses a camera to record the reaction of a series of raised bumps on the back of a fabric film when it comes into touch with an item [118]. This sensor provides feedback on the object’s shape and size by detecting edge information. Furthermore, Guangdong University of Technology has developed a soft hand that incorporates tactile sensing capabilities using a TacTip sensor and several Flex sensors. By using advanced technology, the soft gripper is able to possess exceptional grasping and object detection skills [132]. The University of Bristol conducted further research where they modified the DIGIT by including a 3D-printed sensing surface inspired by the TacTip family of soft biomimetic optical tactile sensors. This modified version is referred to as the DIGIT-TacTip (DigiTac). The tactile sensor possesses more friction, better sensitivity to texture, and the capacity to accurately monitor the test form with submillimeter precision [32]. To validate the applicability of the Tactip sensor in robots, the tactile foot of a legged robot at the University of Bristol, based on the high-resolution bionic TacTip tactile sensor [119], enables it to detect the edge of a beam and the edge of a curved semi-round table and to move the foot to a safe position to safely traverse dangerous areas. The DotTip tactile sensor developed by Zhejiang University is installed on the robot hand. DotTip controls the robot hand to follow the random movement of the experimenter while maintaining the required contact force. In addition, four DotTip tactile sensors are installed on each fingertip of the fully actuated four-finger Allegro, which enables control of the grip force and guarantees success when grasping objects [120]. The sensor has the characteristics of simple manufacture, low cost, and simple application, so it has a wide application prospect in different types of robots.

These sensors (e.g., GelSight and DigiTac) are able to detect small texture and shape changes on the surface of an object, which is especially important in underwater environments. The surface of objects in underwater environments is often covered or blurred by water currents, mud, etc. With these sensors, soft grippers are able to accurately sense the surface characteristics of an object for better gripping and manipulation. In addition, the high-resolution capability of the TacTip sensors can help underwater robots (such as step-footed robots) detect beam edges and curved half-round table edges, ensuring safe robot movement in underwater environments. By providing accurate edge detection and positional adjustment capabilities, these sensors help robots and grippers walk and maneuver safely in complex underwater terrain. The DotTip tactile sensor enables the robot to accurately control the grasping force, ensure the success rate of grasping objects, improve the safety of objects to be grasped, and reduce the possibility of damage to objects.

The above applications of visual and tactile sensors provide a new idea for the realization of the perception of underwater soft grippers, but there is a lack of research on the structural optimization problem of the combination of visual and tactile sensors and underwater soft grippers so as to improve the perception performance and clamping performance of underwater soft grippers. In addition, because the underwater soft gripper needs to work in a complex underwater environment, and the soft film of the vision-tactile sensor on it is easy to deform under the influence of water dynamic characteristics, it is difficult for the vision-tactile sensor to achieve accurate perception in the underwater environment. In addition, changes in the underwater environment will cause the soft film of the visual and tactile sensor to deform, therefore impacting the sensing performance, as shown in Table 3. Therefore, at present, there is a lack of research on the perception algorithm coupling underwater environmental factors with the perception of visual and tactile sensors, which makes it difficult for underwater soft grippers to perceive the complex underwater environment through cross-modal tactile sensors.

### 3.5. Other Tactile Sensors

In addition to tactile sensors based on electrical impulses, magnetic fields, and vision, there are various additional kinds of tactile sensors, such as ultrasonic tactile sensors, nano-tactile sensors, and so on. These kinds of tactile sensors offer the benefits of quick reaction speed, high resolution, and high sensitivity, as illustrated in Table 3.

Southern Taiwan University of Science and Technology has presented an ultrasonic tactile sensor for real-time contact force measurements and high-resolution shape identification to allow safe and accurate robotic grasping of objects that may vary in compliance or texture. The detecting technology applied in this tactile sensor is akin to ultrasonic pulse-echo ranging used in medical imaging or Sound Navigation And Ranging (SONAR) [133]. Increased microstructure density and decreased size resulted in better form identification resolution. However, this leads to lower contact force measurement sensitivity, particularly when the applied force is significant. The University of Massachusetts has designed and fabricated a multidirectional force sensing system based on a flexible sensor for underwater environments, which can efficiently detect and estimate forces from multiple directions [121], but the sensor requires high water resistance. Kagawa University has successfully created a scanner-type measurement instrument with a built-in nano-tactile sensor for monitoring live creatures and skin surfaces. The device with a built-in nano-tactile sensor for measuring living organisms and skin surfaces has a maximum displacement resolution of less than 42 nm and an input resolution of less than 12 µN, enabling the acquisition of frictional force and irregular changes on the target surface with a sensitivity greater than that of a fingertip [122]. However, its production is difficult, costly, sensitive to environmental conditions and external interference, and typically needs extensive signal processing.

Saeed Akbarzadeh et al. presented a novel design and development of a low-cost and multi-touch sensor based on capacitive variations. The experiment showed that the sensors measured the applied forces and contact points with good approximation. The proposed sensor covers a large surface area of the gripper, which is very useful in soft robot grippers for detecting several contact points [123]. V. Carluccio et al. presented the design of a novel magnetic-based tactile sensor to be integrated into the robotic hand of the humanoid robot Vizzy [124]. In addition, since the aquatic environment is more complex and the degree of interference is more visible, the perception accuracy is considerably challenged. In order to consider the impact of underwater factors on the perception performance of tactile sensors, Zhang Jianjun et al. designed a MEMs-based tactile force sensor, which adopted a silicon cup differential pressure structure, combined with an STM32 data acquisition circuit and a BP network data fusion algorithm solving the cross-sensitivity issue induced by water-liquid pressure in underwater tactile force measurement. It reduces the impact of hydrostatic pressure and increases the measurement accuracy and stability but its structural size is vast and it is difficult to incorporate in the soft gripper.

Li Sen et al. designed a Structured Electronic Skin (SES) to achieve high-sensitivity pressure detection and high-fidelity haptic mapping on complex non-expanding surfaces by preparing 3D electrode arrays via 3D printing and combining them with a molded functional shell (made of ion-conductive silicone rubber) [127]. The design solves the problem of traditional e-skins being difficult to fully conform to coverage on non-expanding surfaces (e.g., spherical surfaces, fingertips, etc.) and provides higher accuracy of haptic sensing and a wider range of applications. In the experimental results, the SES device demonstrated a sensitivity of up to 3.59 nF/kPa/cm^2^, capable of detecting a single Pascal’s pressure of 8.6 Pa, with a mechanical response time of tens of milliseconds, and an adjustable measurement range of up to 500 kPa. In addition, the SES achieves a distribution of 46 haptic sensing units on the 3D printed fingertip, allowing for blind-free pressure detection on complex surfaces and high-precision haptic mapping [125]. From the experimental results, it can be seen that the e-skin haptic sensor features good compliance, high sensitivity and accuracy, and adaptability. However, the design has high manufacturing complexity and material limitations. Yang Gao et al. designed a hydrogel microphone for covert underwater listening [128].

The microphone forms a cavity-free sensor by embedding silver nanoparticles (Ag MNPs) into a hydrogel matrix for high-sensitivity reception of low-frequency acoustic waves from long distances. Unlike conventional dielectric capacitors or cavity-based microphones, this hydrogel device responds to external stimuli through transient modulation of the Electrical Double Layer (EDL), resulting in ultra-high sensitivity without the use of any signal amplification tools. Experimental results show that the hydrogel microphone’s response performance at low frequencies (20 Hz to 600 Hz) is 30 dB better than that of commercial hydrophones. Its Electrical Double Layer capacitance reaches 217 nF/kPa at a bias voltage of 1 V, which is thousands of times more sensitive than that of conventional capacitive sensors. The hydrogel microphone demonstrates high directional sensitivity and is able to detect sound waves from different angles. In addition, its wide frequency response allows for efficient detection of underwater sound waves up to 2 kHz. For static loads, the hydrogel microphone exhibits more than a fourfold change in relative capacitance at a pressure of 5.4 kPa, whereas the device without metal nanoparticles performs poorly [126]. However, their pressure response is limited, and their structural complexity is high. In addition, He Qingsong et al. designed a wearable strain sensor based on Polyvinyl Chloride (PVC) organo-ionic gel that is highly stretchable, reproducible, and easy to prepare [129]. However, the material properties of this sensor are difficult to regulate, and different DBA and IL contents affect the sensitivity, linearity, and conductivity of the ionogel, requiring precise blending to optimize the performance. Moreover, the recovery rate is limited, and high ionic liquid content may hinder the recovery of the sensing material, affecting stability in long-term use. In addition, there are certain energy dissipation and hysteresis effects during repeated stretching operations that may affect accuracy in some applications.

## 4. Application Fields for Underwater Soft Grippers

In addition to the extensive application possibilities on land, the underwater use of soft robots has also been given increased attention in recent years. Soft grippers are an important application field of soft robotics, and they have gained a lot of attention for their flexibility and adaptation to diverse items and their ability to gently grab delicate or oddly shaped objects underwater [134,135]. To evaluate the state-of-the-art designs, Table 4 lists the performance parameters of underwater soft grippers across representative studies.

**Figure 6:**
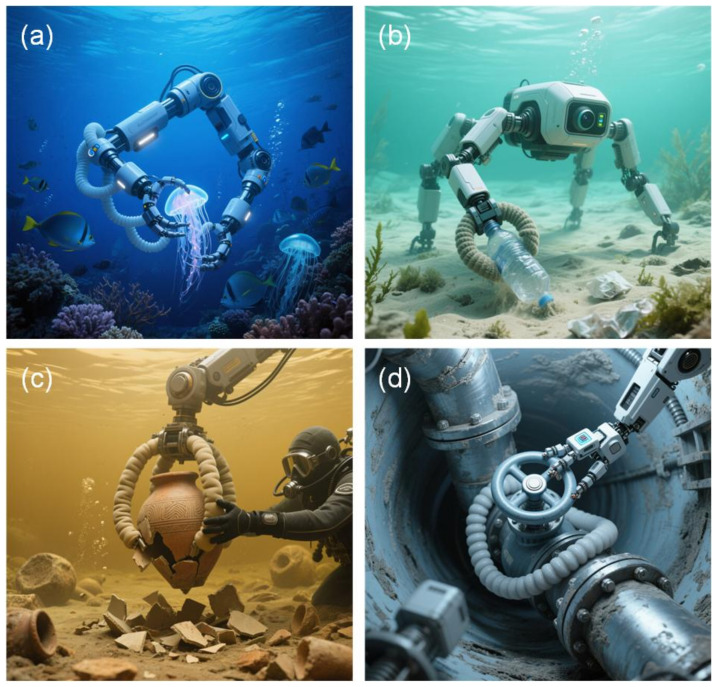
Application of underwater soft grippers. (**a**) Marine research and exploration. (**b**) Submarine garbage pickup. (**c**) Underwater archeology. (**d**) Pipeline operation.

(1) Marine research and exploration [13]: Due to the great flexibility, safety, and powerful adaptation of the underwater soft gripper, the soft gripper is considerably better than the rigid gripper when gripping underwater animals of varied forms [134,135]. In the exploration of the ocean, the underwater soft gripper can sense the geometric aspects of the gripped items, so that the kinds of objects can be identified and categorized in the process of grasping, and the efficiency of the operation can be increased. Figure 6a shows underwater soft grippers in marine research. Their flexibility, safety, and adaptability make them better than rigid ones for grasping various underwater animals, aiding identification via sensing object geometry.
(2) Environmental protection [136]: In recent years, with the development of the ocean and the expansion of the human footprint, there is more and more garbage in the ocean [137]. Part of the garbage is deposited in the seabed, and a soft gripper can have better collection efficiency for garbage with different shapes [138]. Most of the soft underwater robots are propelled by an air pressure or line drive [138], which may decrease environmental contamination and indirect harm to the subsurface environment while operating underwater. Figure 6b depicts an underwater soft robot with a flexible gripper retrieving seabed waste (e.g., plastic bottles), enabling efficient marine debris collection via shape-adaptive grasping.
(3) Underwater archeology [139]: The size and structure of the soft robot may be built to be extremely tiny, and because of its great flexibility, it can penetrate confined spaces to grip things without destroying the site. Figure 6c illustrates a soft gripper retrieving a fragile ancient pot underwater, enabling gentle, damage-free artifact collection at archeological sites via its flexibility.
(4) Underwater rescue: In underwater rescue operations, it is frequently essential to cope with many challenging conditions, such as rescuing trapped persons and moving objects. The flexibility and adaptability of soft grippers make them better suited to tackle these problems and execute accurate clamping and operation to increase rescue efficiency and success rates.
(5) Underwater engineering and maintenance: Underwater soft grippers may be utilized for undersea oil and gas pipeline repair [140], the installation and maintenance of offshore engineering equipment, and other operations. They can grasp and control soft pipes, cables, valves, and more, providing engineers with the flexibility to perform accurate operations and repairs in underwater conditions. Figure 6d shows a soft gripper precisely operating a valve in an underwater pipeline, utilizing flexibility for adaptive manipulation in a confined space.

**Table 4:** Performance parameters of underwater soft grippers.

Application Field	Adaptability	Driving Mode	Stability	Clamping Range	Reference
Marine research and exploration	Tolerates uncertainty of sample size, shape, and stiffness.	Hydraulic drive	The sample can be safely grasped in 100–170 m of water.	It wraps around objects as small as 12 mm in diameter.	[13]
	Work can be performed in a working area with an aperture of 14 cm.	Pneumatic drive	It can work continuously underwater for more than 6 h.	—	[134]
	It can realize the non-destructive grasp of gel-like marine organisms.	Pneumatic drive	The parts will not be damaged by seawater immersion during the service life cycle, and the soft-grip materials have good low temperature properties.	It can grab marine gelatinous creatures of all shapes and sizes.	[135]
Environmental protection	Modular design; it can meet the requirements of various object clamping scenes.	Wire drive	It has good mechanical properties and can hold heavier objects.	—	[138]
Underwater archeology	An experiment involving grasping 107 kinds of objects has been successfully completed, and tasks can be completed in different environments.	Wire drive	It can withstand a pressure of 50 bar and can be used normally after being subjected to pressure.	It can hold objects as small as a coin.	[139]
Underwater engineering and maintenance	Two-way rotation degrees of freedom; can adjust the clamping angle to adapt to different target attitudes.	Pneumatic drive	The sensor feedback closed-loop system can be adjusted in real time according to the grasping state, reducing the risk of sliding.	It can grasp objects with a diameter of 12.5–75 mm, commonly used small tools can be used for better grip.	[140]

In addition to the application areas listed above, the underwater soft gripper can also be used in connection with other institutions or underwater vehicles to boost the efficiency of underwater vehicles. The considerable adaptability and versatility of the soft gripper enable it to be utilized with other kinds of robots or equipment to complete more complex tasks. By merging with other institutions or underwater vehicles, the underwater soft gripper may give full play to its advantages of adaptation and compliance and increase the operating efficiency and flexibility of underwater vehicles. This collaborative method may be extended to a range of underwater missions, enabling improvements in underwater scientific research, engineering, and exploration. With the continuing growth of technology, it is predicted that the collaborative application of underwater soft grippers and other robots will have more innovations and breakthroughs.

## 5. Discussion

Although bionic underwater soft grippers have unique advantages in the fields of marine research and exploration, underwater archeology and underwater rescue, they have certain limitations in the aspects of biomimetic structure, grasping and sensing coupling structure design, and underwater grasping and sensing performance. These include the following:

1. Although many bionic soft grippers have been designed, the structure of biomimetic soft grippers for jellyfish and sea anemones is less, and for biomimetic soft grippers such as sea anemones, the capture characteristics, movement characteristics, and structural structure of sea anemones have not been integrated with the underwater gripper to develop a gripper solution suitable for underwater environments.
2. Although many studies have shown that the application of a tactile sensor provides a new idea for the realization of the perception of a soft gripper, there is a lack of research on the integrated structural design of the combination of a tactile sensor and an underwater gripper so as to improve the sensing performance and clamping performance of the underwater gripper.
3. The use of a tactile sensor in air exhibits distinct benefits. However, there are few investigations on the underwater tactile sensor so far; therefore, there is a dearth of study on the perception algorithm connecting the underwater ambient elements with the tactile sensor perception. As a consequence, it is difficult for the underwater soft gripper to comprehend the complex underwater world via the touch sensor.
4. Due to the great complexity of underwater environments, the underwater soft gripper has certain limits for the gripping of underwater items. In terms of material durability and biofouling, materials used for soft gripping, such as silicone or other elastomers, may wear out and degrade over time due to prolonged exposure to salt water, UV radiation, and biological growth (biofouling). These factors can affect the flexibility, strength, and function of the grip.

Future research should explore advanced coatings or new materials that can resist these environmental factors. The control system is susceptible to sensor noise and delay. The precise control and sensing required to operate effectively in complex underwater environments remains a major challenge. Water currents, pressure variations, and changing lighting conditions can affect the accuracy and responsiveness of the sensors and actuators of soft grippers. It is difficult to accurately describe the complex fluid–structure coupling effect in modeling. Improved algorithms for adaptive control and robust sensing techniques are needed to increase the reliability of these systems. In terms of energy efficiency and power supply, underwater soft robots, including grippers, often face challenges related to energy efficiency and power supply. Battery life limitations and difficulties in transmitting power in underwater environments may limit the operating time and capabilities of these grippers. The development of more energy-efficient actuators and innovative power solutions, such as harvesting energy from the environment, can provide practical solutions. And the effect of underwater elements (water flow, water pressure, etc.) on gripping performance is largely handled via structural design and materials. In addition, the materials that are used to make the grippers have deficiencies in durability and resistance to biological adhesion. Therefore, due to the lack of a method to construct the grasping force compensation mechanism under the action of underwater factors and a material with good biological adhesion and high durability, the grasping force of the soft grippers is unstable, making the soft gripper unable to grasp objects safely in the underwater environment.

In general, there are numerous challenges that remain to be addressed in the future research of bionic underwater soft grippers. For example, although the bionic structure can improve the flexibility and adaptability of the gripper, it also increases control complexity. The underwater environment has high requirements regarding the sealing performance of the devices. If the sealing performance is insufficient when working underwater, the devices may fail, which affects the safety and reliability of underwater operations. In addition, the manufacturing material of the underwater soft gripper will also affect its working stability, and the underwater environment is complex and changeable, which puts forward higher requirements for the corrosion resistance, wear resistance, and anti-aging properties of the material.

However, there are many potential benefits of bionic underwater soft grippers in the future. For example, flexible materials and bionic design enable the underwater soft gripper to reduce the damage to the grasped object and protect the ecological environment and biological resources during operation. At the same time, the soft clamp can also reduce mechanical shock and vibration during operation and improve the safety of the operator. In addition, the research and development of underwater soft grippers needs to integrate technology from multiple disciplines, including material science, mechanical engineering, bionics, sensor technology, etc. This interdisciplinary cooperation will promote the continuous innovation and development of related technologies and bring more breakthroughs to underwater robot technology.

## 6. Conclusions

This paper systematically reviews the technological evolution of soft grippers, with a particular focus on the design of soft grippers inspired by marine organisms such as octopuses, jellyfish, and sea anemones. By summarizing the comprehensive synergy strategies of their structural topology and driving mechanisms, it analyzes the correlation between bionic morphological features and functional realization. Furthermore, this paper reviews the principal architectures of current tactile sensing technologies and critically assesses the limitations of various sensing modes based on their adaptability to underwater applications: electrical signal sensing is susceptible to disturbances from the dielectric constant and conductivity of water; magnetic field sensing is sensitive to changes in water conductivity; and visual sensing is vulnerable to fluid dynamics disturbances and water pressure. In response to these challenges, a “Grasping-Perception Integration” system integration solution is proposed, aiming to enhance the closed-loop control performance of underwater soft gripper systems through the collaborative optimization of embedded sensing and actuation units. The research also distills the core difficulties in the design of underwater soft grippers: optimizing control redundancy (reducing system complexity), ensuring the reliability of fluid sealing (resisting high-pressure penetration), enhancing material environmental durability (resisting corrosion/biofouling), stabilizing dynamic grasping force (suppressing fluid–solid coupling disturbances), and improving multi-modal sensing capabilities (adapting to underwater environments). Overcoming these bottlenecks is a necessary condition for achieving the highly robust operation of underwater soft gripper systems and represents an important research direction for future underwater soft grippers. It will also provide transformative technical support for major application scenarios such as deep-sea resource exploration, underwater emergency rescue, and marine ecological monitoring.

## Figures and Tables

**Table 2:** Bionic categorization and benefits and downsides of bionic soft underwater gripper.

Items	Biological Characteristics	Grasping Mode	Advantage	Disadvantage	Grasping Item Scale	Grasping Force	Driving Mode	References
Octopus-inspired underwater soft gripper	The tentacles feature many suckers and a delicate body.	Crimp, adsorption grab	Excellent stability, flexibility, and adaption	High control complexity, high sealing performance requirements	Maximum grab size: 99 mm; minimum grab size 20 mm	Maximum grasping force: 46 N; minimum grasping force: 3.1 N	Pneumatically driven/cable driven	[66,67,68,69,70,71,72]
Jellyfish-inspired underwater soft gripper	The mouth portions are towards the bottom of the colloidal body, and the tentacles encircling them are radial.	Covering grab	High adaptability, solid grasp	Susceptible to environmental factors	—	Maximum grab weight: 135.3 g; minimum grab weight 59.5 g	Pneumatically driven	[15,73]
Sea anemone-inspired underwater soft gripper	The cylindrical or conical body has rings of overlapping tentacles loaded with stinging cells.	Swallowing, multi-tentacle grab	Strong environmental adaptation, strong gripping success, easy fine object grasping	Complex, hard for designing and build	Maximum grab size: 10 mm; minimum grab size: 4 mm	The gripped item weighs 1 g	Photo/pneumatically driven	[74,75,76]
Other bionic soft grippers	—	Curl, multi-finger grasp	Steady grab, can grab delicate things, good anti-interference ability	Poor adaptability and limited durability	Maximum grab size: 200 mm; minimum grab size: 3 mm	The lifting weight can reach up to 20 kg and the minimum weight is 114.31 g	Pneumatically/hydraulically driven	[13,16,38,57,77,78,79,80,81,82]

## Data Availability

The data and materials that support the findings of this study are available from the corresponding author upon reasonable request.
